# Characterization of Archaeological Artefacts Using Methods Specific to Materials Science: The Case Study of Dacian Ceramics from 2nd c. BC to 1st c. AD

**DOI:** 10.3390/ma14143908

**Published:** 2021-07-13

**Authors:** Laura Teodorescu, Ayed Ben Amara, Nadia Cantin, Rémy Chapoulie, Cătălin Ducu, Sorin Ciucă, Claudiu Tulugea, Carol Terteci, Mărioara Abrudeanu

**Affiliations:** 1 Institut de Recherche sur les Archéomatériaux—Centre de Recherche en Physique Appliquée à L’archéologie, IRAMAT-CRP2A, UMR5060 CNRS—Université Bordeaux Montaigne, CEDEX 33607 Pessac, France; ayed.ben-amara@u-bordeaux-montaigne.fr (A.B.A.); ncantin@u-bordeaux-montaigne.fr (N.C.); chapouli@u-bordeaux-montaigne.fr (R.C.); 2 Department of Materials Science and Engineering, University of Pitesti, 110040 Pitesti, Romania; catalinducu@yahoo.com (C.D.); abrudeanu@gmail.com (M.A.); 3 Department of Materials Science and Engineering, University Politehnica of Bucharest, 060042 Bucharest, Romania; sorin.ciuca@upb.ro; 4 “Aurelian Sacerdoțeanu” Vâlcea County Museum, 240096 Râmnicu Vâlcea, Romania; claudiu.tulugea@yahoo.com (C.T.); terteci.carol@gmail.com (C.T.); 5 Department of Materials Science and Engineering, Technical Sciences Academy of Romania, 030167 Bucharest, Romania

**Keywords:** Dacian archaeological ceramic, materials science, mineralogy, petrography, cathodoluminescence, XRD, SEM–EDX

## Abstract

Combined analysis methods such as optical microscopy (OM), cathodoluminescence (CL) microscopy, X-ray diffraction (XRD), and scanning electron microscopy–energy dispersive X-ray spectrometry (SEM–EDX) have made it possible to obtain the first physico-chemical data of Dacian potsherds, exhumed at the archeological site of Ocnița-Buridava, Romania; the samples were provided by the “Aurelian Sacerdoțeanu” County Museum Vâlcea, dating from the 2nd century BC to the 1st century AD. The mineralogical and petrographic analyses revealed two types of ceramic pastes, taking into account the granulometry of the inclusions and highlighting the choice of the potter for fabricating the ceramic either by wheel or by hand. All samples showed an abundance in quartz, mica (muscovite and biotite), and feldspars. These observations were confirmed by cathodoluminescence imagery, revealing heterogeneous pastes with varied granulometric distributions. The XRD patterns indicated the presence of the mineral phases, indicating a firing temperature below 900 °C. The wheel-made ceramics have a fine, compact matrix with very fine inclusions (<40 µm). On the other hand, the hand-made ceramics present a coarse matrix, with inclusions whose granulometry reaches approximately 2 mm. The difference between these two types of ceramics is also confirmed by the mineralogical and chemical analysis. The wheel-made potsherds are more abundant in MgO, Al_2_O_3_, and CaO contents.

## 1. Introduction

Pottery and, more precisely, ceramic artefacts are one some of the most studied objects by archeologists, since they can be found in large amounts in the majority of archeological sites, dating from the Neolithic period onwards. Therefore, the study of these objects has been essential to the archeological interpretation of a site, region and period. The analytical techniques that have been developed in the field of materials science are widely applied to the study of the ancient objects of art and archaeology, in order to obtain information about the composition and structure of the material used [1,2,3].

One of the main aspects in approaching the study of archaeological materials is to properly identify the type of material that is being investigated, which is the first step in interpreting how it was made, its field of use, its origin, and other cultural information related to the human community. Archaeometric methods involve the integration of exact sciences, especially physics, chemistry, and materials science, in the study of artifacts. It should be taken into account the fact that each analytical technique offers advantages but also limitations, so a broad vision is needed to understand the obtained results [4].

The main aim of this ongoing research is to determine the chemical and mineralogical characteristics of ceramics and to see if it is possible to discriminate ceramics according to their manufacturing technique [5]. These datasets allow the formulation of hypotheses on the nature of the raw materials used and the heat treatments applied. The first aspect focuses on the petrographic analysis (imagery-data) of ceramic fragments. These analyses provide information such as the mineralogical composition of the clay sediment used in the formation of ceramics. The images are then be correlated with the images acquired by the cathodoluminescence. The second aspect focuses on X-ray diffraction in order to determine the mineral content of the potsherds. The results are then compared with petrographic observations to confirm the presence of certain minerals/phases. Finally, the third aspect focuses on the analysis with SEM–EDX. It provides information about the chemical composition of major and minor elements, as well as information about the micro-texture of the ceramics.

## 2. Materials and Methods

### 2.1. Archaeological Context and Potsherd Descriptions

In 1973, excavations were carried out by D. Berciu’s team from the Institute of Archaeology in Bucharest in the area of the archeological site of Ocnița; fragments of ceramic, metal, and glass objects were discovered [6,7,8]. The Dacian city was of great importance to the region; however, after the Roman conquest, the Dacian city began to lose its prestige [9]. Archaeological discoveries have highlighted the role and status of the Dacian settlement in trade with the Roman world, as well as the existence of a possible center of dynastic power. The area is well known for its richness in salt since prehistory. The Latin inscriptions, the numerous Roman artifacts, and the chronology give Buridava a special status, even during the 1st century BC [10,11,12].

**Figure A1:**
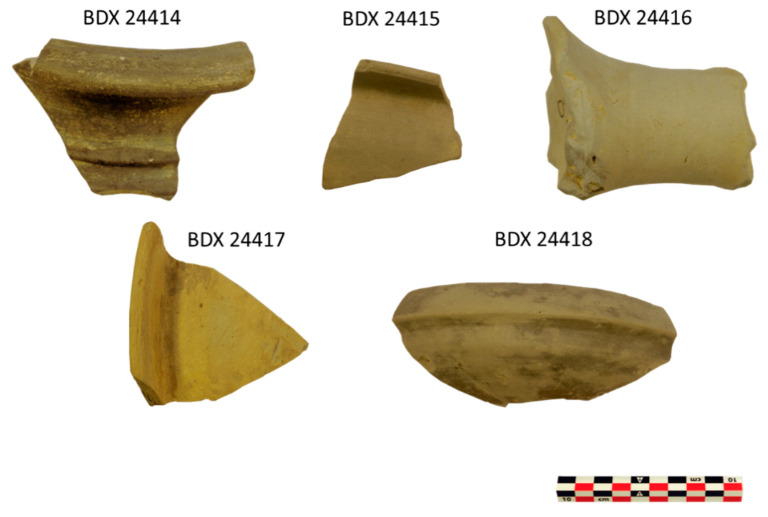
Description of ceramic fragments made by wheel, donated by the museum of Rm. V âlcea “Aurelian Sacerdo*ț*eanu”, Vâlcea County History Museum. The wheel was used at all stages of the manufacturing process.

**Figure A2:**
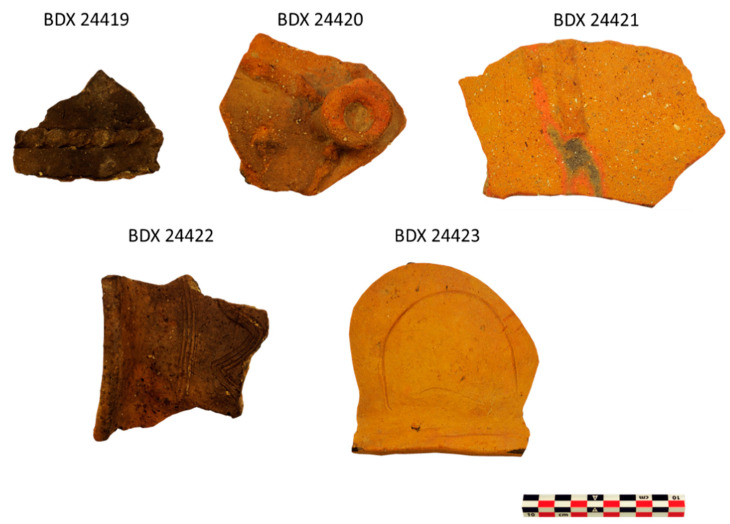
Description of ceramic fragments made by hand, donated by the museum of Rm. Vâlcea “Aurelian Sacerdo*ț*eanu”, Vâlcea County History Museum.

In this study, 10 different ceramic fragments were selected (Table 1). They come from incomplete vessels, discovered during the excavations, dating from the La Tène culture. The archeologists provided five wheel-made potsherds Figure A1) and five hand-made potsherds (Figure A2) for our research. They selected the samples based on macroscopic observations (morphology and topography of the surface and fresh-fractures), the color of the ceramic body, granulometry, and the proportion of inclusions corresponding to their manufacturing technique. The samples were indexed at Bordeaux laboratory (IRAMAT-CRP2A), from BDX 24414 to BDX 24423. Two types of samplings were made per ceramic fragment in order to prepare on one hand powder and on the other, thick and thin sections.

### 2.2. Petrographic Analysis on Thin Sections

Petrographic analysis (OM) was used to characterize non-plastic inclusions in ceramic paste, especially minerals and rocks [13,14]. The inclusions were identified, as well as the shape and particle size distribution. The repartition and shape of the pores were also taken into account. The study was performed on thin sections orientated perpendicular to the vessel wall (thickness 30 μm) using a polarizing microscope (Leica Microsystems, Wetzlar, Germany) coupled to a LEICA DM2500 camera (Leica Microsystems, Wetzlar, Germany). LEICA Application Suite V3 software was used to acquire and record the images. The images were transmitted in parallel and cross-polarized light. For the description of thin ceramic sections, F.J Pettijohn’s diagrams were used to assess the particle size distribution and pore shape [15].

### 2.3. Cathodoluminescence (CL) on Thick Sections

Cathodoluminescence (CL) (i.e., the emission of photons in the visible wavelength range of the electromagnetic spectra under cathode excitation) is a very helpful method used for the study of ceramics [16]. When the surface of a material is bombarded by the beam of electrons, the result is a photon emission in the spectrum (near UV-IR visible) [17,18,19]. For analysis, the Cathodyne OPEA equipment updated (Microvision Instruments, Evry, France) has a cold cathode cathodoluminescence system, paired with a Leica M125 binocular magnifier and a Leica DFC4500 digital camera (Leica Microsystems, Wetzlar, Germany) to capture images (using LAS software). The electron beam was fixed and positioned at 45° to the surface of the part. The exposure time for CL images was about 15 s with a current density of 10 µA. It is a non-destructive analysis regarding the integrity of the ceramic material.

### 2.4. X-ray Diffraction (XRD) on Powder

The XRD measurements were performed on powder to identify the minerals in the ceramic body, using a diffractometer (D8-Advance, set in Bragg–Brentano reflection mode, Bruker AXS, Karlsruhe, Germany) and an X-ray tube with Cu Kα radiations operating at 40 kV and 40 mA. The measurements were recorded from 3° to 60°, with a scan step size of 0.01° and an acquisition time step of 1 s. Qualitative analysis of the obtained diffractograms was realized with EVA software using the PDF-2004 database from ICDD. In addition, a Rietveld refinement was applied by using TOPAS software, in order to quantify the mineral phases.

### 2.5. SEM–EDX on Pellets and Thick Sections

SEM imagery (JEOL-IT500 HR, JEOL Ltd., Tokyo, Japan) made it possible to observe the micro-texture of the samples on thick polished sections. The microscope was used in low-vacuum mode with a pressure of 30 Pa. The parameters used for the analysis of the samples were an acceleration voltage of 20 kV and a probe current from 10^−10^ to 5 × 10^−9^ A. Micrographs were recorded in back-scattered electron mode (BSE). The acquisition of the spectra (performed with a double EDX, Oxford Instruments UltimMax 100, Oxford Instruments, Oxford, UK) was done on the clay matrix and powder pellets. The chemical composition was obtained by quantification from the average of 4 areas of 0.58 mm^2^ each. All results were expressed in wt.% oxides. Data obtained in low-vacuum mode offer results equivalent to those acquired in high-vacuum mode [20]. Quantification was determined using the ϕ (ρz) correction procedure for the AZtec NanoAnalysis (Oxford Instrument, Oxford, UK). This analysis made it possible to quantify major and minor elements such as Na_2_O, MgO, Al_2_O_3_, SiO_2_, SO_3_, K_2_O, CaO, TiO_2_, MnO, and Fe_2_O_3_. Standard corrections were performed using the software’s internal standard. Contents were calculated from standards consisting of synthetic compounds and natural minerals. The detection limit for most elements was about 0.1 wt.%. It addition, the composition data were converted into a log ratio analysis, which made it possible to explore the interdependent relationships in the case of a multivariate dataset and to focus on the covariance and correlation between the variables [21,22].

## 3. Results

### 3.1. Petrographic Analysis

Information about the microstructure of ceramics is first obtained by analysis under a polarizing microscope. Petrographic analyses were performed on all 10 thin sections.

The petrographic analysis made it possible to observe the granulometry and the distribution of the inclusions, allowing us to make initial assumptions about the manufacturing techniques. Microscopically, all the ceramic bodies are mainly composed of quartz, feldspars, micas (biotite and muscovite), amphiboles, and voids (pores).

**Figure 1:**
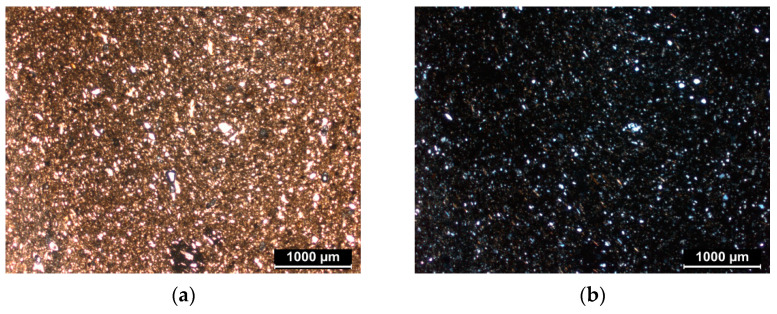

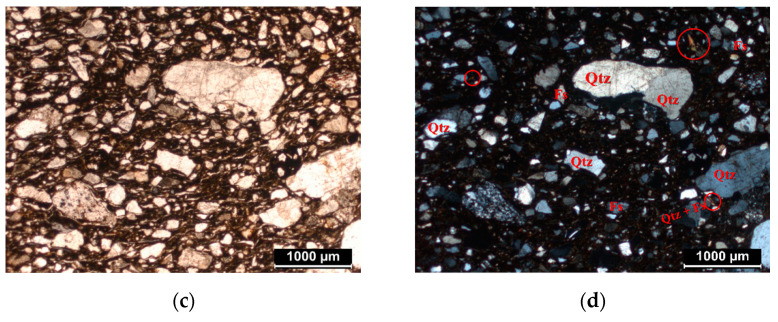
Petrographic observations: observation on matrix of BDX 24418 ((**a**) plane-polarized light, (**b**) cross-polarized light); observation of matrix of BDX 24419 ((**c**) plane-polarized light, (**d**) cross-polarized light). The identified minerals are mainly quartz (Qtz), altered feldspars (Fs) and mica (indicated by the circle).

The wheel-made samples (from BDX 24414 to BDX 24418) show a fine and compact matrix. All these samples are very rich in very fine inclusions, which do not exceed 40 µm in size (Figure 1a,b). The size distribution of the non-plastic components presents a unimodal texture. It can be assumed that the clay raw material has been specially purified or selected. The presence of voids is low, demonstrating the compactness of the ceramic. The porosity seen under the microscope indicates a controlled and slow burning in special furnaces, such as the two-chamber furnace discovered at Buridava Dacică [23,24].

In contrast, the hand-made samples also contain metamorphic rocks and acid plutonic rocks. They present a coarse matrix, with inclusions whose granulometry reaches approximately 2 mm (Figure 1c,d). The size distribution of the present inclusions present a trimodal texture and the porosity observed is mainly represented by elongated voids. Usually, these voids are the primary pores, randomly distributed in the ceramic body. They are formed during the modelling process, when thin layers of water and/or air are trapped between the layers of clay. Then, after the drying and burning of the ceramic, the pore size primarily increases.

The difference in porosity typology between the samples produced by wheel and by hand may be the result of the variation of the molding technique.

Figure 1 shows images acquired with the polarizing microscope, highlighting the difference between a very fine matrix and a coarse one (with the same scale—1 mm).

### 3.2. Cathodoluminescence Imaging

The first results from cathodoluminescence made it possible to formulate hypotheses regarding the nature of the minerals in the clay matrix.

In the wheel-made samples, a compact ceramic body was observed, with low luminescent inclusions with yellow-green and blue color. As noticed in the petrography, the presence of the very fine and few inclusions provided evidence for the purified clay used for manufacture by wheel, which reflects the choice of the potter for obtaining a plastic clay.

**Figure 2:**
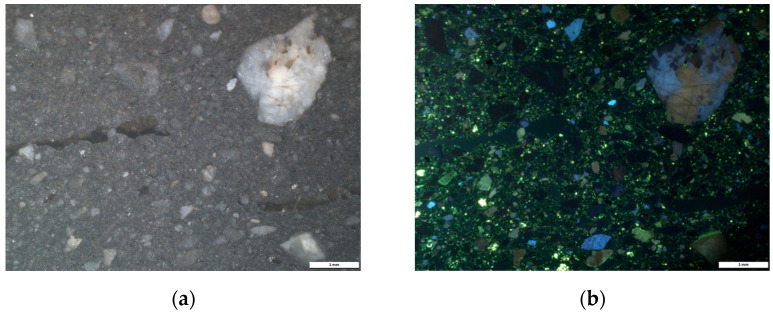

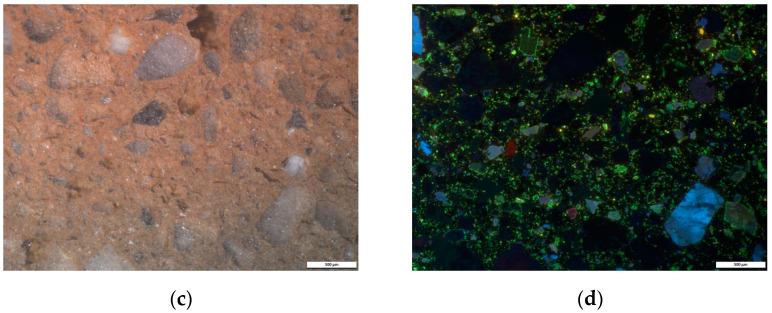
Observations in CL-imagery for ceramic fragments made by hand: sample BDX 24419 ((**a**) white light reflectance, (**b**) cathodoluminescence emission); and sample BDX 24420 ((**c**) white light reflectance, (**d**) cathodoluminescence emission).

For the hand-made samples, the CL images (Figure 2) show the presence of large angular inclusions (from approximately 1 mm to 2 mm) distributed in the matrix. Porosity was observed and identified in the CL images as dark green areas.

As in the wheel-made samples, the main colors seen in CL were a blue luminescent color highlighting the presence of potassium feldspars, the yellow-green luminescence representing plagioclases, and a brownish luminescent color combined with non-luminescent minerals, which represent the quartz inclusions (Figure 2) [25].

In addition, since no inclusions with orange or red luminescent colors where observed in any of the samples, inclusions that are usually characterized as calcium carbonates [26] allowed us to make initial assumptions, such as the clay used by potters being a Ca-poor clay.

### 3.3. XRD Analysis

The X-ray diffraction analysis presented some differences considering the abundance or deficiency of the mineralogical phases (Table 2).

Initially, we noted a certain variability in each manufacturing-technique group, which can be seen in the high values of the standard deviation. In addition, we noted mineralogical differences between wheel-made and hand-made ceramics, in agreement with the petrographic analyses.

In all the samples, reflections of muscovite/illite were detected, although two samples from the wheel-made sample set (BDX 24415 and BDX 24416) showed lower values than the others. The quartz content is globally higher in the hand-made ceramics. In the wheel-made samples, higher values of microcline and anorthite were noted compared with the hand-made samples (Table 2).

It should be noted that illite-muscovite can be used as a thermal guide. Based on previous studies, Rodriguez-Navarro [27] explained that the muscovite crystals can face a significant change from heating at temperatures above 350 °C. Furthermore, previous studies have demonstrated the fact that illite (muscovite) is completely decomposed at about 900 °C [28,29]. In addition, the anorthite detected in all the samples did not result from a transformation at a high temperature during newly formed Ca phases, but rather, from a natural presence in the clayey sediments used for the manufacture of the ceramics. To summarize, in all the samples, the firing temperature was relatively low (below 900 °C), which is in agreement with their porosity aspects.

### 3.4. SEM–EDX Analysis

The SEM observations revealed the microtexture of the potsherds made by wheel and by hand. In addition, SEM–EDX analyses were performed on both the ceramic powder and thick sections.

**Figure 3:**
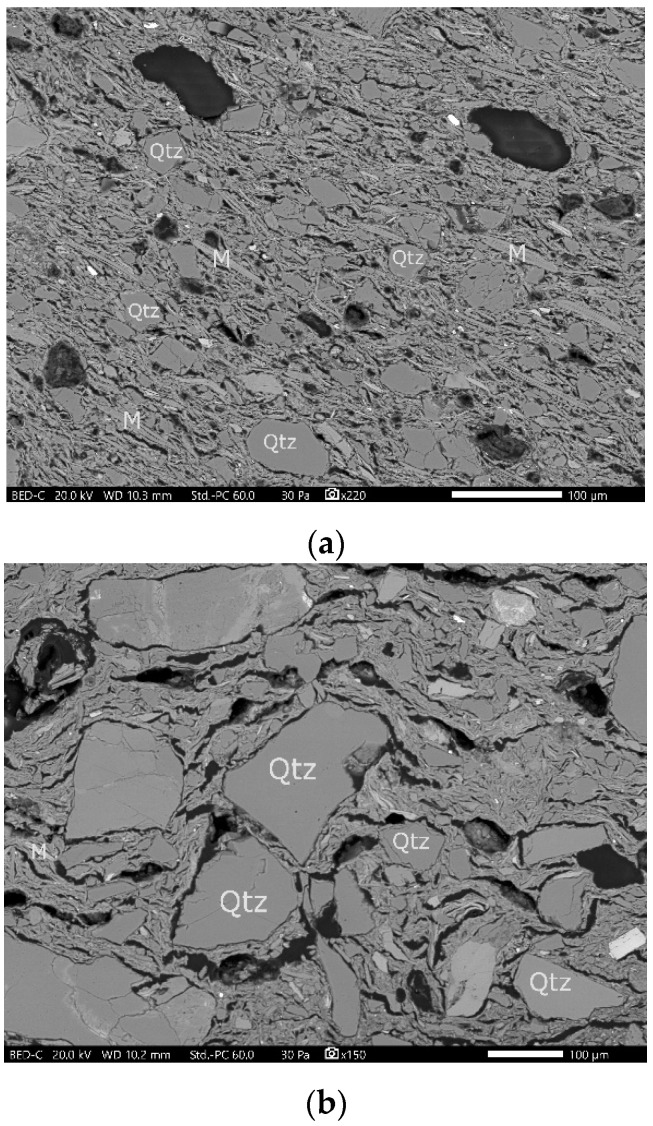
BSE SEM images showing quartz (Qtz) and mica (M) inclusions: (**a**) sample BDX 24414, scale 100 μm; (**b**) sample BDX 24421, scale 100 μm.

The SEM images revealed a compact matrix and the presence of fine inclusions present in the potsherds made by wheel, confirming the observations by petrography and cathodoluminescence. In addition, regarding the size of the inclusions, a clear difference was observed corresponding to the two manufacturing-technique groups (Figure 3).

Due to SEM–EDX, the values obtained for the powder samples were expressed in oxide percentages, choosing the average value of the spectra (Table 3).

The matrix of all the samples indicates the following composition: 58–70% SiO_2_, 16–21% Al_2_O_3_, 1–5% CaO, 6–8% Fe_2_O_3_, 3–4% K_2_O, and 1–4% MgO. Therefore, the clay presents as a Ca-poor one, with a dominant alumino-silicate matrix, as observed by petrography and CL. The high calcium content (in comparison to the CaO content from the hand-made ceramics) in the wheel-made ceramics is likely correlated to the anorthite content.

**Figure 4:**
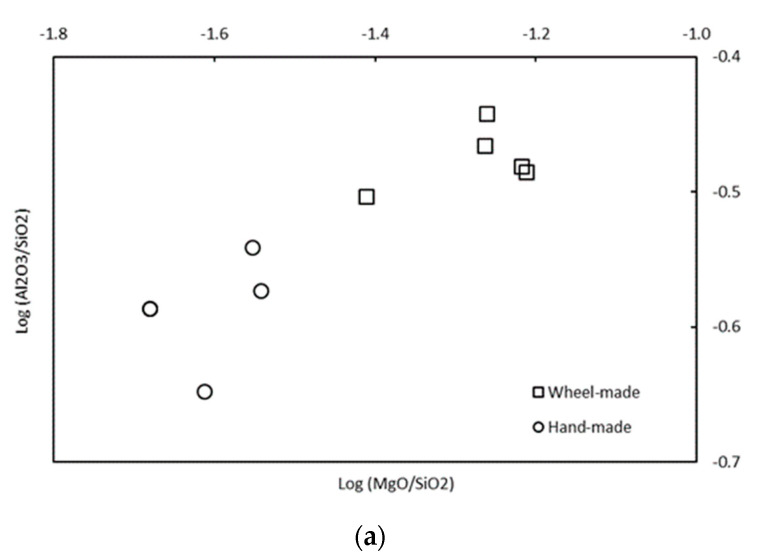

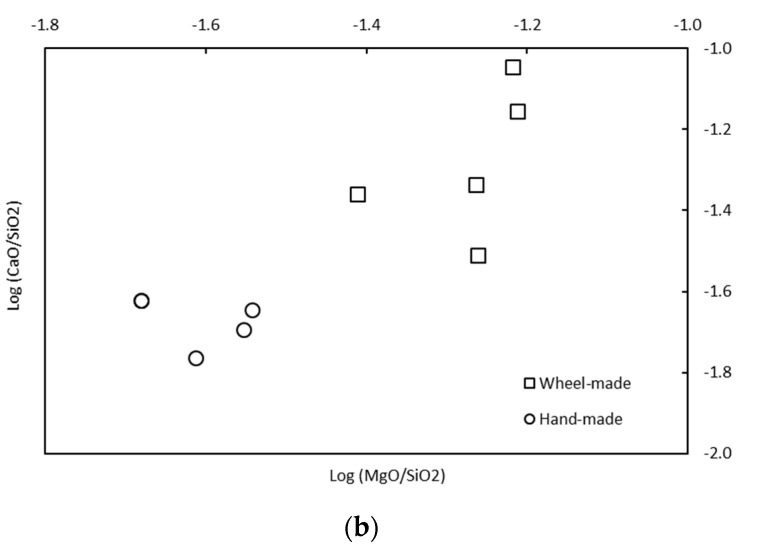
Binary diagrams of potsherds (square-shape potsherds made by hand; circle-shape potsherds made by wheel) expressed in log Al_2_O_2_/Si-log MgO/SiO_2_ (**a**) and log CaO/SiO_2_-log MgO/SiO_2_ (**b**). Hand-made samples BDX 24419 and BDX 24421 present very close values, so their points are overlapping.

Furthermore, it was noted that two groups of chemical composition can be distinguished by the Al_2_O_3_, SiO_2_, CaO, and MgO content (Table 3), in concordance with the manufacture-type groups. The group represented by the potsherds made by wheel is more abundant in MgO, Al_2_O_3_, and CaO contents, with less SiO_2_ content. In order to achieve a better visualization of these two groups, binary diagrams using log-ratios on Al_2_SiO_3_/SiO_2_-MgO/SiO_2_ and CaO/SiO_2_-MgO/SiO_2_ were made (Figure 4). The differences observed between the two groups for microtexture and mineralogy are reinforced by the chemical data. Based on these results, it is most likely that the clay sediments employed are different and were chosen to adapt to the manufacturing technique (more inclusions for the hand-made ceramics).

## 4. Conclusions

Combined analysis methods made it possible to distinguish two types of ceramic manufacturing techniques for ten Dacian potsherds, exhumed at the archeological site of Ocnița-Buridava, Romania, dating from the 2nd century BC to the 1st century AD.

Non-calcareous clay sediments were used to make all the ceramics. We observed a major difference in grain size between the ceramic sherds. Those made by hand are coarser. Petrographic analysis, cathodoluminescence and SEM images show that the proportion of inclusions (mostly quartz, muscovite, and albite) is higher in this group. The granulometric distribution suggests that these non-plastic inclusions were initially present in the clay sediment rather than temper added by the potter. The presence of these inclusions reduces the plasticity of the clays and provides a more suitable raw material for the manufacture of hand-made ceramics.

At this stage of the study, and from the limited number of samples, we have noted differences in terms of texture and chemical and mineralogical compositions between the ceramics according to their manufacturing technique. Fine clay sediments (eventually obtained by removing a large part of the inclusions) were chosen to make the ceramics by wheel, and coarse clay sediments to make the ceramics by hand. For all the ceramics, the firing temperature was relatively below 900 °C, in agreement with their porosity aspects. It is necessary to extend this study to a larger number of samples and to other nearby sites to confirm these initial results; nevertheless, these initial investigations have allowed the generation of archaeometric data that will enable the creation of an initial database for future comparisons when additional data are recorded.

## Figures and Tables

**Table 1:** Detailed context and features of potsherds studied in this research (YD—year of discovery, Tr.—trench number).

Technology Group	Sample ID	Archaeological Context	Category	Object Type
Wheel-made	BDX 24414	YD. 1975, Tr. XXVE	Fine	Pitcher
	BDX 24415	YD. 1976, Tr. XXIX		Fruit bowl
	BDX 24416	YD. 1975, Tr. XXVF		Fruit bowl
	BDX 24417	YD. 1968, Tr. Xa		Bowl
	BDX 24418	YD. 1974, Tr. XXVD		Bowl
Hand-made	BDX 24419	YD. 1975, Tr. XXVF	Coarse	Bowl
	BDX 24420	YD. 1974, Tr. XXVD		Bowl
	BDX 24421	YD. 1968, Tr. Xa		Bowl
	BDX 24422	YD. 1974, Tr. XXVD		Bowl
	BDX 24423	YD. 1975, Tr. XXVF		Bowl

**Table 2:** Mineralogical composition (wt.%) according to X-ray diffraction analysis by Rietveld method. Hematite detected with a value of <1%.

Technology Group	Sample ID	Illite-Muscovite	Biotite	Quartz	Microcline	Orthoclase	Albite	Anorthite	Hornblende
Wheel-made	BDX 24414	22	3	36	6	5	18	9	1
	BDX 24415	11	4	35	20	1	35	14	3
	BDX 24416	11	3	32	16	5	32	14	5
	BDX 24417	31	8	23	9	5	23	12	5
	BDX 24418	21	7	29	17	1	29	11	4
Hand-made	BDX 24419	19	6	43	3	5	13	7	4
	BDX 24420	14	4	55	7	1	9	7	2
	BDX 24421	16	5	43	2	6	20	6	3
	BDX 24422	25	5	37	14	1	6	7	4
	BDX 24423	20	8	35	8	3	13	10	4

**Table 3:** Chemical composition (wt.%) of potsherds obtained by SEM–EDX (SD—standard deviations, number of measurements: n = 4).

Technology Group	Sample ID	Na_2_O	MgO	Al_2_O_3_	SiO_2_	P_2_O_5_	K_2_O	CaO	TiO_2_	MnO	Fe_2_O_3_
Wheel-made	BDX 24414	1.1	2.4	19.4	61.8	1.0	3.3	2.7	0.9	0.1	7.2
	BDX 24415	1.4	3.5	19.1	57.8	0.8	3.5	5.2	0.9	0.1	7.6
	BDX 24416	1.3	3.6	19.2	58.7	0.5	3.6	4.1	1.0	0.1	7.9
	BDX 24417	1.1	3.2	21.1	58.4	2.1	3.6	1.8	1.0	0.1	7.7
	BDX 24418	1.1	3.2	20.1	58.7	1.5	3.6	2.7	1.0	0.1	8.0
	Average	1.2	3.2	19.8	59.1	1.2	3.5	3.3	1.0	0.1	7.7
	SD	0.1	0.5	0.8	1.6	0.6	0.1	1.3	0.1	0.1	0.3
Hand-made	BDX 24419	1.7	1.4	17.4	67.1	1.5	2.5	1.6	0.7	0.2	5.9
	BDX 24420	1.6	1.4	17.4	67.1	1.6	2.5	1.6	0.7	0.2	6.0
	BDX 24421	1.5	1.7	15.7	69.7	0.7	2.6	1.2	0.8	0.1	6.1
	BDX 24422	0.9	1.8	18.5	64.3	1.4	3.5	1.3	0.8	0.2	7.1
	BDX 24423	1.6	1.9	17.7	66.2	0.8	2.7	1.5	0.8	0.1	6.6
	Average	1.5	1.6	17.3	66.9	1.2	2.8	1.4	0.8	0.2	6.3
	SD	0.3	0.2	1.0	1.9	0.4	0.4	0.2	0.1	0.1	0.5

## Data Availability

The data presented in this study are available on request from the corresponding author.
